# Determinants of dental caries among primary schoolchildren in a mountainous region of Northern Vietnam

**DOI:** 10.3389/froh.2025.1675274

**Published:** 2025-10-03

**Authors:** Le Thi Thanh Hoa, Pham Xuan Tue, Hoang Thi Vinh, Truong Thi Thuy Duong, Le Thi Kim Dung

**Affiliations:** 1 Faculty of Public Health, Thai Nguyen University of Medicine and Pharmacy, Thai Nguyen, Vietnam; 2 General Hospital No. 4, Sa Pa, Lao Cai, Vietnam; 3 Vinh Loc District Health Center, Vinh Loc, Thanh Hoa, Vietnam; 4 Department of Pediatrics, Thai Nguyen University of Medicine and Pharmacy, Thai Nguyen, Vietnam

**Keywords:** dental caries, children, Vietnam, DMFT, oral health behavior, mountainous region, school-based intervention

## Abstract

**Background:**

Tooth decay remains one of the most prevalent chronic conditions affecting children globally, especially in underserved populations. Despite growing international attention, limited data exist on the oral health status of children in remote regions of Vietnam.

**Objective:**

To assess factors associated with dental caries among primary schoolchildren in the mountainous province of northern Vietnam.

**Methods:**

A cross-sectional study was conducted from April 2024 to April 2025 among 545 children enrolled in four primary schools in Sa Pa, Lao Cai Province. Clinical dental examinations were performed using the International Caries Detection and Assessment System (ICDAS). Data on oral health behaviors and sociodemographic characteristics were collected via structured interviews. Descriptive statistics summarized the prevalence of caries and oral hygiene practices. Associations were analyzed using stepwise logistic and linear regression models.

**Results:**

The overall prevalence of dental caries was 91.4%, with 57.4% of children affected in permanent teeth and 82.2% in primary teeth. The mean DMFT and dmft scores were 1.31 and 5.38, respectively. Older age increased the risk of permanent caries but reduced primary caries and dmft scores. Significant disparities were observed by ethnicity, with Tay children showing higher odds of primary caries and Hmong children lower dmft scores compared to Dao peers. Children of non-farmer parents, particularly workers, had lower dmft scores than those of farmers. Frequent candy/snack and soda consumption were linked to higher primary caries, while poor brushing habits and symptomatic-only dental visits were associated with worse caries indices.

**Conclusions:**

Children in remote mountainous areas of Vietnam bear an alarmingly high burden of untreated tooth decay, particularly in primary teeth. Sociodemographic factors and inadequate oral health practices significantly contribute to this burden. Tailored, school-based preventive interventions, improved parental education, and enhanced access to dental care are urgently needed to address disparities and improve children's oral health outcomes in these regions.

## Introduction

Tooth decay, or dental caries, remains one of the most prevalent chronic diseases among children worldwide, posing a significant challenge to public health (1). Studies from different regions have consistently shown high and unequal prevalence rates of tooth decay in children. For instance, over 40% of preschool children in rural and remote areas of Western Australia have at least one decayed tooth, with Indigenous children experiencing a significantly higher rate of 69%, compared to 25% among non-Indigenous children (2). Similarly, research in China indicated that 8.7% of 7- to 8-year-olds had caries in their new adult teeth, with plaque detected in over 98% of cases (3). In the United States, 23% of children aged 2–11 years have untreated decay in primary teeth, particularly among low-income and minority groups (4). In Africa, the prevalence among 12-year-olds averages 36%, with large discrepancies between urban and rural settings (5), while in crisis-affected Syria, rates have soared to 79.1% among children aged 8–12 years (6).

Beyond prevalence, the burden of tooth decay manifests in significant health and social consequences. In Western Australia, tooth decay is a leading cause of hospitalization among preschool children, disproportionately affecting Indigenous communities (2). In the United States, untreated caries is associated with pain, infections, and functional impairments, highlighting the importance of preventive programs such as school-based sealants (7). Children with neurodevelopmental disabilities in New Zealand have higher rates of hospital admissions due to dental caries, suggesting the need for targeted interventions (8). Furthermore, research in Tanzania has shown that dental caries negatively impacts preschool children's quality of life, leading to pain, embarrassment, and stigma (9).

A multitude of factors contributes to the development and progression of tooth decay in children. Socioeconomic status is a central determinant, with children from low-income families facing greater challenges in accessing dental care and preventive services (4, 10). Racial and ethnic disparities further exacerbate the issue, especially in the United States, where Hispanic and Black children have higher rates of untreated decay due to systemic and structural barriers (11). Parental factors such as maternal education and infant feeding practices also play a role, as observed in Iran where lower maternal education and prolonged breastfeeding were associated with higher caries scores (12). In addition to socioeconomic and demographic influences, behavioral factors such as dietary habits and oral hygiene practices significantly impact children's oral health. A study in Peru found a strong association between frequent sweet consumption and early childhood caries (13). Regular tooth brushing and the use of fluoride toothpaste are essential preventive behaviors, yet many children fail to adhere to these recommendations, often due to a lack of parental guidance or education (14). Moreover, disparities in access to preventive services remain a major challenge. While interventions such as fluoride varnishes and routine dental visits are proven to reduce caries risk, children from underserved populations continue to face barriers in obtaining these services (4).

Despite the growing body of international evidence, there remains a scarcity of research focused on the oral health of children in remote and underserved regions such as mountainous areas in Vietnam. Limited access to healthcare infrastructure, socioeconomic challenges, and cultural factors may further compound the risk of tooth decay in these populations. Evidence from other regions of Vietnam highlights the scale of the problem: a study conducted in Southern and Central Vietnam found a high prevalence of early childhood caries (ECC) at 74.4% among children aged 1–6 years, with a significant portion of these cases being untreated (15). In Ho Chi Minh City, the prevalence of dental caries among 12-year-olds was influenced by community water fluoridation, with areas having lower fluoride levels showing higher caries rates (16). To address the existing research gap, the present study aims to assess the prevalence and associated factors of tooth decay among schoolchildren living in mountainous regions of northern Vietnam. This research will provide critical insights needed to inform public health policies and targeted interventions for improving oral health equity among vulnerable children.

## Materials and method

### Study design and participants

This study employed a descriptive cross-sectional design and was conducted from April 2024 to April 2025 among schoolchildren enrolled in four primary schools in Sa Pa Town, Lao Cai Province, Vietnam. The target population included all students attending these schools during the survey periodIn Sa Pa Town, there are a total of eight primary schools; four schools were randomly selected to participate in the study. Within each selected school, all classes from grades 1–5 were invited to take part. Eligibility criteria were clearly defined. All children who were present at school during the data collection period and whose parents or legal guardians provided written informed consent were invited to join the study. Exclusion criteria included: (i) children with congenital oral malformations, (ii) those suffering from infectious diseases or fever at the time of the dental examination, (iii) children whose parents or legal guardians declined to give written informed consent, and (iv) children who personally refused to participate. The sample size was calculated based on a formula for cross-sectional studies, using a margin of error (d) of 0.07 and an estimated prevalence (p) of 78.5% derived from a previous study in Malaysia and Myanmar (17). With a 95% confidence level, the required sample size was 532. To account for an anticipated 5% non-response rate, the final adjusted sample size was 559. In practice, 545 students fulfilled the inclusion criteria and consent requirements and were examined, yielding a participation rate of 97.5%.

### Data collection and measurement

Data collection was carried out using two main instruments: a clinical dental examination form and a structured interview questionnaire. The clinical examination was conducted by licensed dentists specialized in Odonto-Stomatology, following a standardized protocol to ensure consistency and reliability. Each child was examined in a designated area within the school premises, under natural or artificial lighting as needed. Oral examinations were performed using sterilized dental instruments, including a mouth mirror and a blunt-tipped explorer, and adhered strictly to the diagnostic criteria of the International Caries Detection and Assessment System (ICDAS) (18). To optimize visibility and enable reliable detection of caries in a non-clinical setting, teeth were dried using sterile cotton rolls and gauze before examination. Only cavitated lesions (corresponding to ICDAS codes 3–6) were recorded in this study, while early enamel lesions (ICDAS 1–2) were not included. All teeth were examined in sequence, and each tooth surface was assessed for signs of cavitated decay. Prior to data collection, examiner calibration was conducted to ensure methodological rigor. The dentists underwent joint training sessions using ICDAS reference photographs and clinical cases, following established guidelines (19). Inter- and intra-examiner reliability were assessed on a subsample of 30 children not included in the main study (19). The kappa coefficients for caries detection ranged from 0.82–0.89, indicating high agreement. In addition to the caries assessment, the Decayed, Missing, and Filled Teeth index was calculated separately for permanent teeth (DMFT) and primary teeth (dmft) to quantify the overall burden of dental caries in the study population.

Sociodemographic and behavioral data were obtained through structured face-to-face interviews using a pre-designed questionnaire. The questionnaire was adapted from previously validated instruments on oral health practices and was translated into Vietnamese (9, 14, 20–22). It was then reviewed by local experts to ensure cultural appropriateness and face validity. A pilot test was conducted with 30 children and their parents/guardians in the study area to confirm clarity, language comprehension, and reliability before implementation in the main survey. The questionnaire collected information on age, gender, school grade, parental occupation, and number of children in the family. The questionnaire also explored understanding of correct oral hygiene methods, such as the appropriate type of toothbrush (child vs. adult), the number of tooth surfaces to brush, the recommended brushing duration and frequency, and suitable brushing times during the day (after meals, before sleep, upon waking). Additional questions evaluated practical behavior, such as how often the child brushed their teeth, when brushing occurred, how often toothbrushes were replaced, and their frequency of consuming sweets or soft drinks. Other behaviors included the use of toothpicks or dental floss, the habit of biting hard objects, and frequency of dental check-ups, ranging from regular preventive visits to visits only when experiencing pain or inflammation. Brushing technique was also directly assessed by observation using a standard toothbrush provided during the interview.

### Statistical analysis

All data were coded and entered using Epidata 3.1, then analyzed with STATA version 17.0. Descriptive statistics (frequencies, percentages, means, and standard deviations) were used to summarize sociodemographic characteristics, oral health status, and knowledge/practices. Associations between independent variables and dental caries outcomes were assessed using stepwise logistic regression (for binary outcomes such as presence of caries) and linear regression (for continuous outcomes like DMFT score). A *p*-value < 0.2 was used as the threshold for selecting variables in the stepwise regression models (23, 24), and *p* < 0.05 was considered statistically significant in the final model.

### Ethical approval

The study protocol was reviewed and approved by the Ethical Review Board of Thai Nguyen University of Medicine and Pharmacy under Decision No. 1197/ĐHYD-HĐĐĐ. Participation was voluntary, with written informed consent obtained from parents or legal guardians. The confidentiality and anonymity of participants were strictly maintained throughout the study. Children found to have oral health issues during the examination were referred for further consultation and management.

## Results

The study included 545 primary schoolchildren, comprising 265 boys (48.6%) and 280 girls (51.4%), with a mean age of 9.1 ± 1.4 years. The largest age group was 10 years old (25.8%), followed by 9 years (22.0%), 11 years (17.8%), 7 years (17.3%), and 8 years (17.1%). Most fathers were farmers (81.7%), while smaller proportions were workers (8.0%), state employees (5.5%), traders (1.9%), or had other occupations (2.9%). Similarly, the majority of mothers were farmers (87.4%), with fewer working as workers (4.7%), state employees (4.7%), traders (1.7%), or in other roles (1.5%). Regarding ethnicity, 42.8% were Dao, 40.7% Hmong, 9.5% Tay, and 7.0% belonged to other groups. In terms of dentition stage, most children were in the mixed dentition phase (90.8%), with 6.3% in permanent dentition and 3.0% still in primary dentition (Table 1).

**Table 1:** Characteristics of participants by gender.

Group	Category	Male	Female	Total
		*N* (%)	*N* (%)	*N* (%)
Total		265 (48.6%)	280 (51.4%)	545 (100.0%)
Age (years)	7 years old	32 (15.4%)	42 (19.2%)	74 (17.3%)
	8 years old	35 (16.8%)	38 (17.4%)	73 (17.1%)
	9 years old	47 (22.6%)	47 (21.5%)	94 (22.0%)
	10 years old	55 (26.4%)	55 (25.1%)	110 (25.8%)
	11 years old	39 (18.8%)	37 (16.9%)	76 (17.8%)
	Mean (SD)	9.2 (1.3)	9.0 (1.4)	9.1 (1.4)
Father's occupation	Farmer	216 (84.1%)	213 (79.5%)	429 (81.7%)
	Worker	19 (7.4%)	23 (8.6%)	42 (8.0%)
	State employee	14 (5.5%)	15 (5.6%)	29 (5.5%)
	Trader	5 (2.0%)	5 (1.9%)	10 (1.9%)
	Other	3 (1.2%)	12 (4.5%)	15 (2.9%)
Mother's occupation	Farmer	228 (88.0%)	237 (86.8%)	465 (87.4%)
	Worker	10 (3.9%)	15 (5.5%)	25 (4.7%)
	State employee	12 (4.6%)	13 (4.8%)	25 (4.7%)
	Trader	4 (1.5%)	5 (1.8%)	9 (1.7%)
	Other	5 (1.9%)	3 (1.1%)	8 (1.5%)
Ethnic group	Dao	109 (41.1%)	124 (44.3%)	233 (42.8%)
	Hmong	113 (42.6%)	109 (38.9%)	222 (40.7%)
	Tay	24 (9.1%)	28 (10.0%)	52 (9.5%)
	Other	19 (7.2%)	19 (6.8%)	38 (7.0%)
Dentition stage	Primary	9 (3.4)	7 (2.5)	16 (3.0)
	Mixed	241 (91.6)	251 (90.0)	492 (90.8)
	Permanent	13 (4.9)	21 (7.5)	34 (6.3)

Table 2 summarizes the dental health status of schoolchildren by gender. The prevalence of permanent tooth decay was 54.0% in males and 60.7% in females, while deciduous tooth decay was observed in 84.5% of males and 80.0% of females. The proportion of children with any tooth decay was similar between genders (91.7% in males and 91.1% in females). The mean number of decayed permanent teeth was slightly higher in females (1.38 ± 1.54) than in males (1.21 ± 1.45), and the DMFT score was 1.38 ± 4.54 in females and 1.24 ± 4.76 in males. For deciduous teeth, males had a higher mean number of decayed (5.49 ± 4.62) and missing (0.22 ± 0.93) teeth compared to females (4.90 ± 4.46 and 0.10 ± 0.48, respectively), while females had a slightly higher mean number of filled deciduous teeth. The dmft index was higher in males (5.74 ± 4.76) than in females (5.04 ± 4.54). Most comparisons did not show statistically significant differences between genders, except for the number of filled permanent teeth (*p* = 0.039).

**Table 2:** Dental health status by gender.

Group	Category	Male	Female	Total	*p*-value
		*N* (%)	*N* (%)	*N* (%)	
Permanent tooth decay	Yes	143 (54.0%)	170 (60.7%)	313 (57.4%)	0.111
	No	122 (46.0%)	110 (39.3%)	232 (42.6%)	
Deciduous tooth decay	Yes	224 (84.5%)	224 (80.0%)	448 (82.2%)	0.167
	No	41 (15.5%)	56 (20.0%)	97 (17.8%)	
Any tooth decay	Yes	243 (91.7%)	255 (91.1%)	498 (91.4%)	0.794
	No	22 (8.3%)	25 (8.9%)	47 (8.6%)	
		**Mean** **±** **SD**	**Mean** **±** **SD**	**Mean** **±** **SD**	*p*-value
Number of decayed permanent teeth		1.21 ± 1.45	1.38 ± 1.54	1.29 ± 1.50	0.124
Number of missing permanent teeth		0.01 ± 0.11	0.00 ± 0.06	0.01 ± 0.09	0.290
Number of filled permanent teeth		0.02 ± 0.19	0.00 ± 0.00	0.01 ± 0.14	0.039
DMFT index		1.24 ± 4.76	1.38 ± 4.54	1.31 ± 4.66	0.179
Number of decayed deciduous teeth		5.49 ± 4.62	4.90 ± 4.46	5.19 ± 4.55	0.119
Number of missing deciduous teeth		0.22 ± 0.93	0.10 ± 0.48	0.16 ± 0.74	0.112
Number of filled deciduous teeth		0.02 ± 0.16	0.04 ± 0.25	0.03 ± 0.21	0.192
Dmft index		5.74 ± 4.76	5.04 ± 4.54	5.38 ± 4.66	0.084

Among participant characteristics, only **age** showed a statistically significant association with dental caries in both permanent and primary teeth. In permanent dentition, the proportion of children with caries increased steadily from 35.1% at age 7%–63.2% at age 11 (*p* = 0.003). For primary dentition, the prevalence of decay was very high across all ages but decreased with increasing age, from 90.4% at 8 years and 86.5% at 7 years to 57.9% at 11 years (*p* < 0.001). No other variables demonstrated significant associations (Table 3).

**Table 3:** Association between participant characteristics and dental caries.

Characteristic	Group	Decay in permanent teeth			Decay in primary teeth		
		Yes	No	*p*	Yes	No	*p*
		*N* (%)	*N* (%)		*N* (%)	*N* (%)	
Age (years)	7	26 (35.1)	48 (64.9)	0.003	64 (86.5)	10 (13.5)	<0.001
	8	43 (58.9)	30 (41.1)		66 (90.4)	7 (9.6)	
	9	49 (52.1)	45 (47.9)		78 (83.0)	16 (17.0)	
	10	67 (60.9)	43 (39.1)		88 (80.0)	22 (20.0)	
	11	48 (63.2)	28 (36.8)		44 (57.9)	32 (42.1)	
Father's occupation	Farmer	247 (57.6)	182 (42.4)	0.748	353 (82.3)	76 (17.7)	0.697
	Worker	27 (64.3)	15 (35.7)		32 (76.2)	10 (23.8)	
	State employee	15 (51.7)	14 (48.3)		26 (89.7)	3 (10.3)	
	Trader	6 (60.0)	4 (40.0)		8 (80.0)	2 (20.0)	
	Other	7 (46.7)	8 (53.3)		12 (80.0)	3 (20.0)	
Mother's occupation	Farmer	270 (58.1)	195 (41.9)	0.917	383 (82.4)	82 (17.6)	0.233
	Worker	15 (60.0)	10 (40.0)		18 (72.0)	7 (28.0)	
	State employee	14 (56.0)	11 (44.0)		20 (80.0)	5 (20.0)	
	Trader	4 (44.4)	5 (55.6)		9 (100.0)	0 (0.0)	
	Other	4 (50.0)	4 (50.0)		8 (100.0)	0 (0.0)	
Ethnic group	Dao	135 (57.9)	98 (42.1)	0.161	193 (82.8)	40 (17.2)	0.193
	Hmong	118 (53.2)	104 (46.9)		175 (78.8)	47 (21.2)	
	Tay	36 (69.2)	16 (30.8)		47 (90.4)	5 (9.6)	
	Other	24 (63.2)	14 (36.8)		33 (86.8)	5 (13.2)	

 Table 4 presents the association between various oral health behaviors and the presence of dental caries among participants. Significant associations were observed for candy/snack consumption and soda consumption with dental caries in primary teeth. Children who frequently consumed candy or snacks had a higher prevalence of caries in primary teeth (90.6%) compared to those who consumed them occasionally (80.2%) (*p* = 0.016). Similarly, frequent soda consumption was associated with markedly higher caries prevalence in primary teeth (96.3%) compared to occasional consumption (80.5%) (*p* = 0.004). No other behaviors demonstrated statistically significant associations.

**Table 4:** Association between oral health behaviors and dental caries.

Group	Category	Decay in permanent teeth			Decay in primary teeth		
		Yes	No	*p*	Yes	No	*p*
		*N* (%)	*N* (%)		*N* (%)	*N* (%)	
Brushing habit	Yes	292 (58.3)	209 (41.7)	0.184	407 (81.2)	94 (18.8)	0.074
	No	19 (47.5)	21 (52.5)		37 (92.5)	3 (7.5)	
Brushing frequency/day	0	10 (52.6)	9 (47.4)	0.570	18 (94.7)	1 (5.3)	0.051
	1	148 (55.9)	117 (44.1)		225 (84.9)	40 (15.1)	
	2	112 (60.2)	74 (39.8)		144 (77.4)	42 (22.6)	
	3	15 (48.4)	16 (51.6)		28 (90.3)	3 (9.7)	
Morning brushing	Yes	216 (56.8)	164 (43.2)	0.471	307 (80.8)	73 (19.2)	0.372
	No	91 (60.3)	60 (39.7)		127 (84.1)	24 (15.9)	
Brushing after lunch	Yes	20 (47.6)	22 (52.4)	0.163	36 (85.7)	6 (14.3)	0.486
	No	287 (58.7)	202 (41.3)		398 (81.4)	91 (18.6)	
Brushing before bed	Yes	147 (60.0)	98 (40.0)	0.345	199 (81.2)	46 (18.8)	0.779
	No	160 (55.9)	126 (44.1)		235 (82.2)	51 (17.8)	
Brushing after dinner	Yes	21 (67.7)	10 (32.3)	0.249	23 (74.2)	8 (25.8)	0.263
	No	286 (57.2)	214 (42.8)		411 (82.2)	89 (17.8)	
Toothbrush replacement time	<3 months	29 (64.4)	16 (35.6)	0.326	32 (71.1)	13 (28.9)	0.096
	3–6 months	14 (77.8)	4 (22.2)		13 (72.2)	5 (27.8)	
	>6 months	24 (63.2)	14 (36.8)		29 (76.3)	9 (23.7)	
	When damaged	121 (56.5)	93 (43.5)		186 (86.9)	28 (13.1)	
	Other	3 (75.0)	1 (25.0)		3 (75.0)	1 (25.0)	
	Don't remember	116 (54.5)	97 (45.5)		172 (80.8)	41 (19.2)	
Candy/snack consumption	Frequent	60 (62.5)	36 (37.5)	0.273	87 (90.6)	9 (9.4)	0.016
	Occasional	251 (56.4)	194 (43.6)		357 (80.2)	88 (19.8)	
Soda consumption	Frequent	36 (66.7)	18 (33.3)	0.150	52 (96.3)	2 (3.7)	0.004
	Occasional	275 (56.5)	212 (43.5)		392 (80.5)	95 (19.5)	
Biting hard objects	Yes	89 (60.1)	59 (39.9)	0.383	118 (79.7)	30 (20.3)	0.353
	No	216 (56.0)	170 (44.0)		321 (83.2)	65 (16.8)	
Toothpick/dental floss use	None	194 (54.5)	162 (45.5)	0.095	296 (83.2)	60 (16.8)	0.521
	Wood toothpick	12 (48.0)	13 (52.0)		20 (80.0)	5 (20.0)	
	Bamboo toothpick	101 (65.6)	53 (34.4)		124 (80.5)	30 (19.5)	
	Dental floss	3 (60.0)	2 (40.0)		3 (60.0)	2 (40.0)	
Brushing skill (observed)	Yes	262 (57.6)	193 (42.4)	0.227	383 (84.2)	72 (15.8)	0.153
	No	17 (47.2)	19 (52.8)		27 (75.0)	9 (25.0)	
Dental check-up timing	Every 3–6 months	1 (100.0)	0 (0.0)	0.245	0 (0.0)	1 (100.0)	0.104
	When cavities/infection	97 (62.2)	59 (37.8)		133 (85.3)	23 (14.7)	
	Never	187 (56.5)	144 (43.5)		269 (81.3)	62 (18.7)	
	Don't remember	25 (48.1)	27 (51.9)		41 (78.9)	11 (21.1)	

Table 5 presents factors associated with tooth decay (any decay in permant and primary teeth), DMFT, and dmft indices. In the analysis of factors associated with permanent tooth decay, age was significantly related: each additional year increased the odds of decay by 26% (OR = 1.26, 95% CI: 1.07–1.49). Soda consumption was also significant, with occasional consumers showing a lower odds of permanent tooth decay compared with frequent consumers (OR = 0.33, 95% CI: 0.13–0.85).

**Table 5:** Factors associated with tooth decay, DMFT and dmft indices.

Variables	Any tooth decay in permanent teeth		Any tooth decay in primary teeth		DMFT		dmft	
	OR	OR	95% CI		Coef.	95% CI	Coef.	95% CI
Gender (Male vs. Female-ref)	1.46	0.94; 2.27	0.62	0.34; 1.14				
Age (per year)	1.26*	1.07; 1.49	0.58*	0.45; 0.75	0.18*	0.07; 0.29	−1.52*	−1.83; −1.21
Ethnic (vs. Dao-ref)								
Hmong			0.72	0.35; 1.46			−1.53*	−2.46; −0.60
Other			2.48	0.34; 18.20			−0.13	−2.06; 1.79
Tay			19.82*	1.23; 320.22			1.06	−0.79; 2.90
Father's occupation (vs. Farmer-ref)								
Worker							−3.37*	−4.98; −1.75
State employee							−0.55	−2.60; 1.50
Trader							−0.57	−3.74; 2.59
Other							−2.61*	−4.80; −0.41
Mother's occupation (vs. Farmer-ref)								
Worker			0.37	0.11; 1.25			−2.36*	−4.20; −0.53
State employee			0.09*	0.01; 0.59			0.16	−2.13; 2.46
Trader							0.71	−2.20; 3.62
Other							3.84	−0.15; 7.84
Brushing habit (No vs. Yes-ref)	0.49	0.18; 1.38					1.80*	0.16; 3.44
Brushing after lunch (No vs. Yes-ref)			0.41	0.13; 1.28				
Brushing after every meal (No vs. Yes-ref)			2.13	0.71; 6.40				
Candy/snack consumption (Occasional vs. Frequent -ref)			0.31*	0.10; 0.94				
Soda consumption (Occasional vs. Frequent -ref)	0.33*	0.13; 0.85						
Observed Brushing skill (Yes vs. No-ref)	0.46	0.20; 1.07						
Dental check-up timing (vs. Every 3–6 months-ref)								
When cavities/infection					0.54*	0.01; 1.08		
Never					0.19	−0.30; 0.68		

* *p* < 0.05.

For primary teeth, older age was associated with a reduced likelihood of decay (OR = 0.58, 95% CI: 0.45–0.75). Among ethnic groups, children of Tay ethnicity had significantly higher odds of primary tooth decay compared to Dao (OR = 19.82, 95% CI: 1.23–320.22). Candy and snack consumption was also significant, as occasional consumers had lower odds of decay compared with frequent consumers (OR = 0.31, 95% CI: 0.10–0.94). Children having mother who were state employees had a lower odds of having primary tooth decay.

Regarding the DMFT index, older age was associated with an increase in score (Coef. = 0.18, 95% CI: 0.07–0.29). The timing of dental check-ups also showed a significant effect: children who sought care only when cavities or infections occurred had higher DMFT scores compared with those who checked every 3–6 months (Coef. = 0.54, 95% CI: 0.01–1.08).

For the dmft index, older age was significantly associated with lower scores (Coef. = –1.52, 95% CI: −1.83 to −1.21). Children of Hmong ethnicity had lower dmft scores than Dao (Coef. = –1.53, 95% CI: −2.46 to −0.60). Father's occupation was significant, with children of workers (Coef. = –3.37, 95% CI: −4.98 to −1.75) and those with fathers in “other” jobs (Coef. = –2.61, 95% CI: −4.80 to −0.41) showing lower dmft scores. Mother's occupation was also associated, with children of workers having lower scores (Coef. = –2.36, 95% CI: −4.20 to −0.53). In addition, brushing habits mattered, as children who did not brush regularly had higher dmft scores (Coef. = 1.80, 95% CI: 0.16–3.44).

## Discussion

This study highlights a significantly high prevalence of dental caries among primary schoolchildren in a remote mountainous region of Northern Vietnam, with over 90% affected. While there were no major gender differences in decay prevalence, the study identified key sociodemographic and behavioral factors associated with DMFT and dmft scores, suggesting multiple pathways for oral health intervention.

The exceptionally high prevalence of tooth decay (91.4%) and elevated dmft index (mean = 5.38) observed in this study reflect a critical public health issue in the mountainous region under investigation. These findings indicate that most children suffer from untreated caries in their primary teeth, which likely impairs their nutrition, development, and quality of life. The DMFT score for permanent teeth (mean = 1.31), though lower, still signifies early onset of decay that remains unaddressed. In the context of remote mountainous areas, such oral health burdens may be exacerbated by geographical isolation, limited availability of dental professionals, lack of fluoridated water, and insufficient oral health promotion activities.

Comparable challenges have been reported elsewhere in Vietnam. A study conducted in Southern and Central Vietnam found a high prevalence of early childhood caries (ECC) at 74.4% among children aged 1–6 years, with a significant portion of these cases being untreated (15). Similarly, in Ho Chi Minh City, the prevalence of dental caries among 12-year-olds was influenced by community water fluoridation, with areas having lower fluoride levels showing higher caries rates (16). These findings emphasize how both geographic and environmental factors, including water fluoride concentration, strongly shape oral health outcomes in Vietnamese children.

The combination of environmental barriers and socioeconomic disadvantages likely contributes to the persistent and severe nature of dental caries seen in this population. This situation is comparable to findings in other disadvantaged and remote settings worldwide. For instance, Bergeron et al. (2020) reported caries rates of 94%–98% among children in remote Andean communities (21). Trimble et al. (2024) noted an 83% rate in rural El Salvador (25), and Min et al. (2024) observed an 87% rate in Myanmar (17). Aimond et al. (2023) reported a high dental caries prevalence of 78.5% among children, with prevalence increasing with age (17). Peters et al. (2022) found that children in rural areas had a higher mean dmft + DMFT score (1.22) compared to urban children (0.96), with an overall caries prevalence of approximately 39% (26). These comparisons underscore that the problem observed in the mountainous area of northern Vietnam is not isolated but aligns with a broader global pattern affecting remote and underserved children. Effective interventions in this setting must be culturally appropriate, geographically accessible, and embedded in school and community structures to ensure sustainable improvements in oral health.

Sociodemographic factors showed significant associations with oral health outcomes in this study. Age was positively associated with DMFT but inversely related to dmft, indicating that as children grew older, caries experience in permanent teeth increased while the burden in primary teeth declined. This pattern aligns with prior research indicating that caries prevalence tends to decline with age due to improved hygiene practices and the natural exfoliation of primary teeth (20, 27). Ethnic differences were also evident, with Hmong children showing significantly lower dmft scores compared to the Dao group, while Tay children had markedly higher odds of primary tooth decay. Such disparities may stem from cultural dietary habits, oral hygiene norms, or uneven access to dental services among ethnic communities—similar to findings from North America, where Indigenous and minority children bear a higher burden of caries compared to their majority counterparts (11, 28). These results underscore the need to consider cultural and contextual differences in designing oral health interventions in ethnically diverse populations.

Parental occupation also influenced oral health outcomes. Children of fathers working as laborers or in “other” occupations had significantly lower dmft scores than those of farmer fathers, suggesting that income diversification or migration-related work may indirectly benefit oral health. This finding contrasts somewhat with global literature, where lower occupational status is often associated with higher caries risk (20, 29). However, in the local context, it is possible that non-farming parents—often engaged in labor migration or trade—may have higher income or better exposure to urban health literacy, enabling greater investment in their children's oral health. Similarly, maternal occupation was strongly associated with caries outcomes, with children of mothers who were workers having lower dmft scores compared to those of farmer mothers (29). These findings highlight the complex interaction between socioeconomic roles, cultural practices, and access to care, emphasizing the importance of tailoring oral health strategies to local demographic profiles.

Behavioral practices were another key determinant. Children who brushed irregularly, particularly those without a brushing habit, had significantly higher dmft scores, underscoring the protective role of consistent oral hygiene. In addition, frequent candy or snack consumption and frequent soda consumption were both strongly associated with higher odds of primary tooth decay, confirming the cariogenic impact of dietary sugar in this population. Furthermore, children with poor observed brushing skills had elevated dmft levels, highlighting that brushing frequency alone is insufficient without proper technique. This aligns with existing literature showing that many children, especially in disadvantaged settings, perform suboptimal brushing and miss critical areas like canine segments (30). These results emphasize the importance of both oral hygiene behaviors and effective brushing skills in preventing early childhood caries.

Patterns of dental care-seeking further shaped oral health outcomes. Children who visited the dentist only when cavities or infections occurred had significantly higher DMFT scores compared to those with regular preventive visits every 3–6 months. This finding reflects the predominance of curative over preventive care in rural settings, where services are scarce and access is hindered by cost and distance. Previous studies confirm that regular dental visits are associated with better oral health (31, 32), particularly when supported by proactive parental involvement and favorable attitudes toward dental care (22). These findings underline the need for school-based preventive programs and community education initiatives tailored to the geographic and socioeconomic realities of mountainous populations.

The findings of this study have several practical implications for oral health policy and practice in mountainous and underserved regions. The extremely high prevalence of tooth decay and elevated dmft and DMFT indices underscore the urgent need for targeted preventive programs. School-based interventions—such as supervised toothbrushing, regular fluoride varnish application, and parental education—should be prioritized in these settings. Culturally sensitive oral health promotion strategies that engage local communities and consider ethnic differences may also improve outcomes. Furthermore, training and deploying community-based oral health workers could help overcome access barriers in remote areas. Addressing socioeconomic determinants such as parental education and occupation is essential for achieving long-term improvements in children's oral health.

This study has several limitations. First, its cross-sectional design does not allow for causal inferences between behavioral or sociodemographic factors and oral health outcomes. The associations observed may be influenced by reverse causation or unmeasured confounders. Second, reliance on self-reported data (e.g., brushing frequency, toothbrush replacement) may introduce recall and reporting bias. Third, although efforts were made to include diverse ethnic groups, the sample may not be fully representative of all children living in remote mountainous regions. Finally, access to advanced clinical diagnostic tools was limited, and although trained examiners were used, some variation in caries detection may still occur.

Despite these limitations, this study has several notable strengths. It is among the first investigations to provide age- and dentition-specific analyses of dental caries in a remote mountainous area of northern Vietnam, addressing an evidence gap in both national and international literature. The study also used a large sample drawn from multiple schools, enhancing its reliability and representativeness within the target region. Moreover, the standardized diagnostic criteria (ICDAS) and examiner calibration ensured methodological rigor, while the integration of both clinical and behavioral data allowed for a comprehensive assessment of caries determinants.

## Conclusion

This study demonstrates that children in remote mountainous areas face an exceptionally high burden of untreated dental caries, influenced by sociodemographic conditions and oral health behaviors. These findings emphasize the urgent need for culturally tailored and geographically accessible oral health interventions to reduce inequalities and improve well-being among vulnerable populations. Future research should employ longitudinal designs to clarify causal pathways, incorporate more representative sampling across different mountainous provinces, and evaluate the effectiveness of school- and community-based preventive strategies such as fluoride supplementation, oral health education, and improved access to dental care.

## Data Availability

The raw data supporting the conclusions of this article will be made available by the authors, without undue reservation.
